# Posterior approach, fracture diagnosis, and american society of anesthesiology class iii–iv are associated with increased risk of revision for dislocation after total hip arthroplasty: An analysis of 33,337 operations from the finnish arthroplasty register

**DOI:** 10.1177/1457496920930617

**Published:** 2020-06-05

**Authors:** Valtteri J. Panula, Elina M. Ekman, Mikko S. Venäläinen, Inari Laaksonen, Riku Klén, Jaason J. Haapakoski, Antti P. Eskelinen, Laura L. Elo, Keijo T. Mäkelä

**Affiliations:** Department of Orthopaedics and Traumatology, Turku University Hospital, University of Turku, Luolavuorentie 2, Turku, 20700, Finland; Department of Orthopaedics and Traumatology, Turku University Hospital, University of Turku, Turku, Finland; Turku Bioscience Centre, University of Turku and Åbo Akademi University, Turku, Finland; Department of Orthopaedics and Traumatology, Turku University Hospital, University of Turku, Turku, Finland; Turku Bioscience Centre, University of Turku and Åbo Akademi University, Turku, Finland; Turku PET Centre, University of Turku, Turku, Finland; National Institute for Health and Welfare, Helsinki, Finland; Coxa Hospital for Joint Replacement, Tampere, Finland; Turku Bioscience Centre, University of Turku and Åbo Akademi University, Turku, Finland; Turku PET Centre, University of Turku, Turku, Finland; Department of Orthopaedics and Traumatology, Turku University Hospital, University of Turku, Turku, Finland

**Keywords:** Total hip arthroplasty, revision, dislocation, American Society of Anesthesiology class, surgical approach, femoral neck fracture, femoral head size

## Abstract

**Background and objective:**

Dislocation is one of the most common reasons for revision surgery after primary total hip arthroplasty. Both patient related and surgical factors may influence the risk of dislocation. In this study, we evaluated risk factors for dislocation revision after total hip arthroplasty based on revised data contents of the Finnish Arthroplasty Register.

**Methods:**

We analyzed 33,337 primary total hip arthroplasties performed between May 2014 and January 2018 in Finland. Cox proportional hazards regression was used to estimate hazard ratios with 95% confidence intervals for first dislocation revision using 18 potential risk factors as covariates, such as age, sex, diagnosis, hospital volume, surgical approach, head size, body mass index, American Society of Anesthesiology class, and fixation method.

**Results:**

During the study period, there were 264 first-time revisions for dislocation after primary total hip arthroplasty. The hazard ratio for dislocation revision was 3.1 (confidence interval 1.7–5.5) for posterior compared to anterolateral approach, 3.0 (confidence interval 1.9–4.7) for total hip arthroplasties performed for femoral neck fracture compared to total hip arthroplasties performed for osteoarthritis, 2.0 (confidence interval 1.0–3.9) for American Society of Anesthesiology class III–IV compared to American Society of Anesthesiology class I, and 0.5 (0.4–0.7) for 36-mm femoral head size compared to 32-mm head size.

**Conclusions:**

Special attention should be paid to patients with fracture diagnoses and American Society of Anesthesiology class III–IV. Anterolateral approach and 36-mm femoral heads decrease dislocation revision risk and should be considered for high-risk patients.

## Introduction

Dislocation is one of the most common reasons for revision surgery after primary total hip arthroplasty (THA) covering 17%–21% of all first-time revisions^1,2^. Dislocation incidence during the first postoperative year after primary THA varies from 2% to 4%^3–5^. The risk of dislocation is highest during the first 3 postoperative months, but dislocations may also occur later^3^. Majority of the dislocations, from 66% to 69%, occur during the first postoperative year^3,6,7^.

Both patient related and surgical factors may predispose to THA dislocation. Posterior approach, poor component positioning, small femoral head size, implant choice, poor repair of soft tissues, and surgeon experience have generally been accepted as risk factors for surgery related dislocations^3,4,6–8^. Patient-related risk factors for dislocation reported in earlier studies have been higher American Society of Anesthesiology (ASA) class, female sex, older age, operative diagnosis, and neurological and cognitive disorders^3,6,7^. In practice, the reason for THA dislocation is often multifactorial and patient specific^9, 10^. Further, dislocation risk after revision surgery is remarkably higher than that after primary THA^10^.

The Finnish Arthroplasty Register (FAR) has been collecting information on THAs since 1980. In earlier data from the FAR from 1996 to 2010, larger femoral head size clearly reduced the risk of dislocation^11^. However, these data included several thousands of large-head metal-on-metal THAs and hip resurfacing arthroplasties (HRAs), which have been abandoned since then due to metal bearing–related complications. The data contents of FAR have also been thoroughly revised in 2014 to include parameters such as surgical approach, body mass index (BMI), ASA class, intraoperative bleeding, and duration of the operation. Post data content revision FAR data on the dislocation risk have not been assessed earlier.

The objective of this study was to determine risk factors for revision for dislocation after primary THA first time in Finland based on the prospectively collected FAR data from 2014 to 2018 with the revised data contents. This is assessed now since some of the used variables have not been available in the FAR earlier.

## Material and Methods

In Finland, all orthopedic units are obliged to provide all information essential for maintenance of the FAR to the Finnish National Institute for Health and Welfare. Dates of death are obtained from the Population Register Centre. Data completeness in primary THA in the FAR has varied from 91.1% to 95.2% during the years 1997–2015^12^. For revision, THA data completeness is 85%^2^. Finland is a relatively small country where registries and the healthcare system are publicly funded with 100% coverage of hospitals. In case of death, patients are censored from the registry, and this information is updated regularly. Since 19 May 2014, all FAR THA data on implant components have been recorded electronically based on barcode reading. The data contents of FAR were also revised in 2014 also to include several new variables. These are surgical approach, BMI, ASA class, intraoperative bleeding, duration of the operation, level of education of surgeon and assistant, mode of anesthesia, intraoperative complications, and previous operations. The end of the follow-up time of the current study was 31 January 2018. Patient’s minimum follow-up time ranged between 0 and 3.5 years. Revisions were linked to the primary operation through a patient-specific personal identification number and laterality. The survival endpoint was defined as revision where any component, including isolated liner exchange, was removed or exchanged due to dislocation. Data from 33,337 unilateral and bilateral THAs performed in Finland between years 2014 and 2018 were extracted from FAR and included in our study (Table 1).

**Table 1.** Demographic data.

	N / mean	% / SD	N / mean revision for dislocation	% / SD
Number of hips	33,337		264	
Age (years)				
⩽55	4507	14	29	11
56–65	8333	25	55	20
66–75	12,399	37	99	38
⩾76	8091	24	81	31
Number of hips available	33,330		264	
Sex				
Female	19,002	57	161	61
Male	14,317	43	103	39
Number of hips available	33,319		264	
ASA class				
I	4013	12	16	6
II	16,117	49	112	43
III–IV	12,567	39	133	51
Number of hips available	32,697		261	
Preoperative diagnosis				
OA	27,965	87	192	76
Fracture	1366	4	33	13
Other	2984	9	30	11
Number of hips available	32,315		255	
Surgical approach				
Anterolateral (modified Hardinge)	6151	19	22	9
Posterior	26,203	80	235	90
Anterior (Smith–Peterson)	298	1	3	1
Number of hips available	32,652		260	
Intraoperative bleeding (mL)				
<500	21,839	70	159	63
⩾500	9542	30	94	37
Number of hips available	31,381		253	
Anesthesia form (compared to all others)				
Epidural	791	2	13	4
Spinal	30,119	76	237	78
General	2532	6	21	7
Nerve block	6	0	0	0
LIA	6237	16	34	11
Number of hips available	32,604		260	
Fixation				
Cementless	18,655	62	133	54
Cemented	3008	10	33	13
Hybrid	6837	23	69	28
Reverse hybrid	1650	5	12	5
Number of hips available	30,150		247	
Bearing				
Metal-on-UHXLPE	12,652	50	132	63
Ceramic-on-ceramic	2786	11	13	6
Ceramic-on-UHXLPE	7063	28	51	24
Ceramized metal-on-UHXLPE	1445	6	3	2
Other	1161	5	11	5
Number of hips available	25,107		210	
Femoral head size (mm)				
28	347	1	3	1
32	7836	24	87	35
36	23,958	74	158	63
>36	311	1	4	1
Number of hips available	32,452		252	

SD: standard deviation; N: number; ASA class: American Society of Anesthesiology classification; OA: primary osteoarthritis; LIA: local infiltrative anesthesia; UHXLPE: ultra-highly cross-linked polyethylene.

Kaplan–Meier analysis was used to estimate the unadjusted cumulative revision probabilities for dislocation, with 95% confidence intervals (CIs). Univariate and multivariable Cox proportional hazards regression models were used to estimate hazard ratios with 95% CIs for first dislocation revision. Proportional hazards assumption of the Cox model was assessed by visual inspection of Kaplan–Meier curves and by using a test based on the scaled Schoenfeld residuals^13^. Since sex did not fulfill the assumption of proportional hazards, it was used as a stratification variable. After stratification, only comparison of ASA class I versus ASA class II in the multivariable model showed minor violation of the proportional hazards according to the Schoenfeld residuals test (p = 0.04). The corresponding Kaplan–Meier plot is available as an Online Appendix 1. However, we decided to present the data as such, not dividing follow-up in different time periods, to make our results easier to comprehend. We also performed a sensitivity analysis for the findings obtained for different surgical approaches using univariate Cox proportional hazards regression analysis in a subpopulation concerning only so-called healthy standard patients (primary osteoarthritis (OA), ASA class I–II, cementless or hybrid THA, metal-on-ultra-highly cross-linked polyethylene (UHXLPE) or ceramic-on-UHXLPE bearing surface, and head size 36 mm). In addition, we assessed how the used surgical approach affected the occurrence of revision due to dislocation among the patients with a diagnosis of femoral neck fracture. The following risk factors were considered as covariates: age group (⩽55, 56–65, 66–75, ⩾76 years), sex, diagnosis (primary OA, fracture, other), hospital volume (low, medium, high), surgical approach (posterior, anterolateral, anterior), head size (28, 32, 36, >36 mm), BMI (<25, 25–30, >30 kg/m^2^), ASA class (I, II, III–IV), fixation method (cementless, cemented, hybrid, reverse hybrid), previous operation to the same joint like osteotomy or osteosynthesis (yes, no), level of education of the surgeon (specialist, resident), level of education of the first assistant (specialist, resident, other), bleeding (<500, ⩾500 mL), duration of the operation (minutes), anesthesia form (spinal, epidural, general), local infiltrative anesthesia (LIA; yes, no), perioperative complication during surgery (no complication, calcar fracture, trochanteric fracture, femoral shaft fracture, acetabular fracture), bearing surface used (ceramic-on-ceramic, ceramic-on-UHXLPE, metal-on-UHXLPE, ceramized metal-on-UHXLPE, other), and use of oblique liner (yes, no). The classification of the hospitals to the different volume groups was based on the average number of primary THAs performed annually during the study period: less than 240 (low), 240–480 (medium), and more than 480 (high). The number of hips available for analyses for each variable is presented in Table 1 and Online Appendix 2, so the number of missing values can be seen. Only patients without any missing data for variables of interest (N = 21,706) were included in the final multivariable models. All statistical analyses were carried out using R version 3.4.2 (R Development Core Team, http://www.r-project.org). Implant survival was analyzed using R package *survival*^14^. The level of significance was set at p < 0.05.

## Results

In the present study, we analyzed data from 33,337 THAs performed in Finland between years 2014 and 2018 (Table 1). Largest age group in terms of performed primary THA were the patients from 66 to 75 years (37%). Majority of the study population was women (19,002; 57%). Most of the patients had an ASA class II (49%) or combined III and IV (39%) and received a THA with cementless fixation (62%) and with a metal-on-UHXLPE (50%) or ceramic-on-UHXLPE (28%) bearing surface. The main reason for primary THA was primary OA (87%) and the most common surgical approach was posterior approach (80%; Table 1). The overall Kaplan–Meier survival revision for dislocation as the endpoint at 3.5 years was 98.9% (CI: 98.8–99.1).

Posterior surgical approach was significantly associated with increased risk of revision for dislocation when compared to the anterolateral approach in both univariate analysis (hazard ratio (HR) 2.6 (CI 1.7–4.1, p < 0.001); Table 2) and in multivariable analysis (HR 3.1 (CI 1.7–5.5, p < 0.001); Table 3). The anterior approach was not associated with dislocation revision in univariate analysis (HR 2.9 (CI 0.9–9.6), p = 0.09; Table 2), but in multivariable analysis, the anterior approach had an increased risk of revision (HR 3.6 (CI 1.0–13.1), p = 0.05; Table 3). In the sensitivity analysis, HR for posterior compared to anterolateral approach for dislocation revision was 2.1 (CI 0.7–5.8, p = 0.2). Also, THAs performed for femoral neck fracture had an increased risk of revision for dislocation when compared to THAs performed for primary OA in univariate analysis (HR 3.6 (CI 2.5–5.2, p < 0.001); Table 2) and in multivariable analysis (HR 3.0 (CI 1.9–4.7, p < 0.001); Table 3). Patients who received THA for other reasons were not associated with dislocation revision in univariate analysis (HR 1.5 (CI 1.0–2.1), p = 0.05; Table 2) or in multivariable analysis (HR 1.4 (CI 0.9–2.2), p = 0.2; Table 3).

**Table 2.** Univariate analysis of possible predictors for revision for dislocation.

	Hazard ratio	95% CI	p value
Age (years)			0.05
⩽55	Reference		
56–65	1.0	0.7–1.6	0.9
66–75	1.2	0.8–1.9	0.3
⩾76	1.6	1.0–2.4	0.04
ASA class			<0.001
I	Reference		
II	1.8	1.0–3.0	0.03
III–IV	2.7	1.6–4.5	<0.001
Surgical approach			<0.001
Anterolateral (modified Hardinge)	Reference		
Posterior	2.6	1.7–4.1	<0.001
Anterior (Smith–Peterson)	2.9	0.9–9.6	0.09
Femoral head size (mm)			0.002
28	0.8	0.2–2.4	0.7
32	Reference		
36	0.6	0.5–0.8	<0.001
>36	1.1	0.4–3.1	0.8
Preoperative diagnosis			<0.001
OA	Reference		
Fracture	3.6	2.5–5.2	<0.001
Other	1.5	1.0–2.1	0.05
Intraoperative bleeding (mL)			
<500	Reference		
⩾500	1.3	1.0–1.7	0.04
Anesthesia form (compared to all others)			
Epidural	2.0	1.2–3.6	0.01
Spinal	0.8	0.5–1.3	0.3
General	1.1	0.7–1.7	0.7
LIA	0.6	0.5–0.9	0.02
Bearing			<0.001
Metal-on-UHXLPE	Reference		
Ceramic-on-ceramic	0.4	0.2–0.7	0.003
Ceramic-on-UHXLPE	0.7	0.5–1.0	0.03
Ceramized metal-on-UHXLPE	0.2	0.1–0.6	0.006
Other	0.9	0.5–1.6	0.7
Fixation			0.02
Cementless	Reference		
Cemented	1.6	1.1–2.4	0.01
Hybrid	1.4	1.1–1.9	0.01
Reverse hybrid	1.4	0.8–2.5	0.3
Hospital volume			0.06
Low	Reference		
Medium	1.3	1.0–1.8	0.08
High	1.4	1.0–1.9	0.03

CI: confidence interval; ASA class: American Society of Anesthesiology classification; OA: primary osteoarthritis; LIA: local infiltrative anesthesia; UHXLPE: ultra-highly cross-linked polyethylene.

**Table 3.** Statistically significant predictors for revision for dislocation in the multivariable analysis.

	Hazard ratio	95% CI	p value
ASA class			0.09
I	Reference		
II	1.7	0.9–3.3	0.09
III–IV	2.0	1.0–3.9	0.04
Surgical approach			<0.001
Anterolateral (modified Hardinge)	Reference		
Posterior	3.1	1.7–5.5	<0.001
Anterior (Smith–Peterson)	3.6	1.0–13.1	0.05
Femoral head size (mm)			0.004
28	0.5	0.1–3.4	0.4
32	Reference		
36	0.5	0.4–0.7	<0.001
>36	0.4	0.0–2.6	0.3
Preoperative diagnosis			<0.001
OA	Reference		
Fracture	3.0	1.9–4.7	<0.001
Other	1.4	0.9–2.2	0.2
Bearing			0.1
Metal-on-UHXLPE	Reference		
Ceramic-on-ceramic	0.6	0.3–1.3	0.2
Ceramic-on-UHXLPE	0.9	0.6–1.3	0.5
Ceramized metal-on-UHXLPE	0.3	0.1–1.0	0.06
Other	0.6	0.2–1.3	0.2

CI: confidence interval; ASA class: American Society of Anesthesiology classification; OA: primary osteoarthritis; UHXLPE: ultra-highly cross-linked polyethylene.

Only patients without any missing data for variables of interest (N = 21,706) were included in the final multivariable models.

Patients with higher ASA class had significantly increased risk of revision for dislocation in univariate analysis (ASA II versus ASA I, HR 1.8 (CI 1.0–3.0, p = 0.03) and ASA III–IV versus ASA I, HR 2.7 (CI 1.6–4.5, p < 0.001)) and in multivariable analysis (ASA III–IV versus ASA I, HR 2.0 (CI 1.0–3.9, p = 0.04); Tables 2 and 3). In the multivariable analysis, ASA class II compared to ASA class I was not significant (HR 1.7 (CI 0.9–3.3, p = 0.09); Table 3).

The use of 36-mm femoral head size decreased the risk of revision for dislocation compared to 32-mm head in univariate analysis (HR 0.6 (CI 0.5–0.8, p < 0.001); Table 2) and in multivariable analysis (HR 0.5 (CI 0.4–0.7, p < 0.001); Table 3). We found no association between the risk for dislocation revision and the use of other head sizes (28 mm and >36 mm) in univariate analysis (28 mm versus 32 mm, HR 0.8 (CI 0.2–2.4, p = 0.7) and >36 mm versus 32 mm, HR 1.1 (CI 0.4–3.1, p = 0.8)) or in multivariable analysis (28 mm versus 32 mm, HR 0.5 (CI 0.1–3.4, p = 0.4) and >36 mm versus 32 mm, HR 0.4 (CI 0.0–2.6, p = 0.3); Tables 2 and 3).

We found a significantly increased risk of revision for dislocation in univariate, but not in multivariable analysis for the following parameters: high hospital volume versus low hospital volume, intraoperative bleeding ⩾500 mL versus <500 mL, the use of epidural anesthesia, and cemented or hybrid fixation versus cementless fixation (Table 2). There was a significantly decreased risk of revision for dislocation in the univariate but not in the multivariable analysis for the following: the use of LIA; and ceramic-on-ceramic, ceramic-on-UHXLPE, or ceramized metal-on-UHXLPE versus metal-on-UHXLPE (Table 2).

The demographics of the used surgical approaches and the occurrence of revision due to dislocation among the patients with femoral neck fracture diagnosis are described in the Table 4. There were dislocation revisions only among patients who had been operated using posterior approach (Table 4). Therefore, we were not able to perform further statistical analyses on subject.

**Table 4.** The used surgical approaches and the occurrence of revision due to dislocation among patients with femoral neck fracture diagnosis (N = 1366).

Characteristic	Total number of patients with preoperative femoral neck fracture			Number of revisions due to dislocation			Number of patients without subsequent dislocation		
	N available	N	%	N available	N	%	N available	N	%
Number of hips	1366			33			1333		
Surgical approach	1341			33			1308		
Anterolateral (modified Hardinge)		247	18		0	0		247	19
Posterior		1083	81		33	100		1050	80
Anterior (Smith–Peterson)		11	1		0	0		11	1

Data on all tested variables can be found from Online Appendix 2 to 4.

## Discussion

Dislocation is still one of the main reasons for revision operation after primary THA^2,15,16^. We used FAR data from 2014 to 2018 to assess risk factors for dislocation revisions after the primary THA and found that in our material, posterior approach, fracture diagnosis, and ASA class III–IV increased dislocation revision risk when compared to anterolateral approach, primary OA diagnosis, and ASA class I. In addition, in our study, femoral head size 36 mm had decreased dislocation revision risk compared to head size 32 mm.

We found that posterior approach was associated with increased risk for dislocation revision compared to anterolateral approach. Similar results have also been found in previous studies^7,17,18^. In the Dutch Arthroplasty Register, revision for dislocation risk has been from 0.5 to 0.6 for the straight lateral, anterolateral, and anterior approaches while when compared to posterior approach^8^. A Norwegian register study found 2.1-fold risk for dislocation revision for posterior approach compared to the anterolateral approach^18^. It has previously been suggested that patients belonging to risk groups should be operated using lateral approaches^7^. Our results support this proposal. Anterior approach had an increased risk of revision due to dislocation compared to the anterolateral approach in the current study, but the total amount of THAs performed using anterior approach was very small. In sensitivity analysis, the difference of the dislocation revision rate between posterior and anterolateral approach was no longer statistically significant. Sensitivity analysis included approximately 21% of all operations included, so lower power may be the reason for the non-significant result. Anterior and posterior approaches have been associated to have better patient-reported outcome measures compared to anterolateral and direct lateral approaches. Patients operated on posterior approach had less postoperative pain in Numeric Rating Scale (NRS) pain scores during the activity and in rest compared to patients operated on anterolateral approach^19^. In the present study, there were dislocation revisions only among patients with preoperative femoral neck fracture diagnosis who were operated on posterior approach. This finding is consistent with that of prior studies^20–22^.

The Australian registry has reported two times higher and the Swedish registry four times higher dislocation revision risk for patients whose THA was operated due to femoral neck fracture compared to patients who were operated due to OA^7, 23^. Our results are in accordance with these registry findings with threefold dislocation revision risk for THA operated due to femoral neck fractures compared to those operated for primary OA. Special attention on implant choice and approach should be followed when treating fracture patients.

Another factor associated with increased dislocation revision risk in our multivariable model was ASA class III–IV compared to ASA class I. A previous study stated that patients with an ASA class of II or higher had an increased risk of dislocation in the Dutch Register^8^. In our data, ASA class II was a risk factor only in univariate analysis, but otherwise our results support the findings from the Dutch Register. Patients with increased ASA class have more comorbidities and are more fragile which might predispose them for dislocations. In addition, threshold to operate these patients may be higher, and therefore, the primary situation may already be more demanding which might increase the dislocation risk.

Large femoral head size has been previously associated with a decreased risk of revision for dislocation. Based on FAR data on 42,379 THAs and HRAs, the use of 28-mm femoral heads has been reported to have 10-fold dislocation revision risk compared to the >36-mm femoral heads^11^. However, this previous study included several thousand large head metal-on-metal THAs and HRAs and, therefore, is not directly comparable to the current study, which did not include any metal-on-metal bearings. In previous studies, the dislocation revision risk has been reported to be equal for 32- and 36-mm heads^7, 11^. A large registry study conducted by the Nordic Arthroplasty Registry Association from 2003 to 2014 found no difference between 36- and 32-mm heads in relation to dislocation revision risk^24^, contrary to our current finding of lower risk with 36-mm heads. A recent report from the Dutch Arthroplasty Register stated that 36-mm heads reduced the risk of revision for dislocation compared to 32-mm heads, although this finding considered only THAs performed from the posterior approach^8^. Based on these most recent data, 36-mm femoral heads should be considered instead of 32-mm heads for patients with high dislocation risk.

A study of 192,275 THAs from Australia found a higher risk of revision for dislocation for the 36-mm femoral heads with the metal-on-XLPE bearing compared to ceramic-on-cross-linked polyethylene, and ceramic-on-ceramic bearing surfaces^25^. Based on our research, bearing surface material was not, at short term, associated with the dislocation revision rate. Further, oblique liners intended to prevent dislocations did not reduce dislocation revision risk compared to conventional liners in our study. However, we did not assess oblique liners implant wise. It is possible that there are individual products which are effective in this respect. Further research is needed to assess the possible dislocation preventive effect of oblique liners.

Previous literature has presented multiple other factors possibly associated with dislocation risk. One study from the New Zealand registry found lower dislocation revision risk for cemented implants^26^. Even though majority of the studies have not found any association between age and dislocation risk^7,11,23^, contradictive data also exist^27^. Relationship between sex and dislocation rate has as well been conflicting in earlier literature^7,23^. In our data, sex and age did not have significant associations with dislocation revision risk in either univariable or multivariable analysis. Fixation type and hospital volume were associated with dislocation revision in the univariate analysis; however, these differences diminished in the multivariable model. Based on our data, intraoperative bleeding, mode of anesthesia, duration of the operation, level of education, previous operations, or intraoperative complications were not associated with the dislocation revision rate, and we are not aware of any opposite findings.

We acknowledge that our study has several limitations. Comorbidity data of the patients were not available, although ASA class presents a crude estimate of medical condition. In addition, we were unable to assess radiographs and implant positioning. Further, we did not have data on closed repositions of dislocated THA. It is possible that some patients have suffered one or two dislocation and their hip has stabilized after that without a revision operation.

In conclusion, posterior approach compared to anterolateral approach, fracture diagnosis compared to primary OA, and ASA class III–IV compared to ASA class I were associated with increased risk for dislocation revision. Head size 36 mm was associated with decreased revision risk compared to 32-mm heads. These factors should be taken into consideration, especially while treating patients with increased dislocation risk.

## Supplemental Material

sj-pdf-1-sjs-10.1177_1457496920930617 – Supplemental material for Posterior approach, fracture diagnosis, and american society of anesthesiology class iii–iv are associated with increased risk of revision for dislocation after total hip arthroplasty: An analysis of 33,337 operations from the finnish arthroplasty register Click here for additional data file. Supplemental material, sj-pdf-1-sjs-10.1177_1457496920930617 for Posterior approach, fracture diagnosis, and american society of anesthesiology class iii–iv are associated with increased risk of revision for dislocation after total hip arthroplasty: An analysis of 33,337 operations from the finnish arthroplasty register by Valtteri J. Panula, Elina M. Ekman, Mikko S. Venäläinen, Inari Laaksonen, Riku Klén, Jaason J. Haapakoski, Antti P. Eskelinen, Laura L. Elo and Keijo T. Mäkelä in Scandinavian Journal of Surgery

## References

[1] AOANJRR: Annual report 2017. Available at: https://aoanjrr.sahmri.com/fi/annual-reports-2017 (2017, accessed 20 September, 2018).

[2] Finnish Arthroplasty Register (FAR). Available at: https://thl.fi/far/#index (2017, accessed 20 September 2018).

[3] Meek RMD Allan DB McPhillips G , et al: Late dislocation after total hip arthroplasty. Clin Med Res 2008;6(1):17–23. 18591373 10.3121/cmr.2008.770 PMC2442025

[bibr4-1457496920930617] Lübbeke A Suva D Perneger T , et al: Influence of preoperative patient education on the risk of dislocation after primary total hip arthroplasty. Arthritis Rheum 2009;61(4):552–558. 19333972 10.1002/art.24340

[5] Seagrave KG Troelsen A Malchau H , et al: Acetabular cup position and risk of dislocation in primary total hip arthroplasty. Acta Orthop 2017;88(1):10–17. 27879150 10.1080/17453674.2016.1251255 PMC5251254

[6] Werner BC Brown TE : Instability after total hip arthroplasty. World J Orthop 2012;3(8):122–130. 22919568 10.5312/wjo.v3.i8.122 PMC3425631

[bibr7-1457496920930617] Hailer NP Weiss RJ Stark A , et al: The risk of revision due to dislocation after total hip arthroplasty depends on surgical approach, femoral head size, sex, and primary diagnosis. An analysis of 78,098 operations in the Swedish Hip Arthroplasty Register. Acta Orthop 2012;83(5):442–448. 23039167 10.3109/17453674.2012.733919 PMC3488169

[8] Zijlstra WP De Hartog B Van Steenbergen LN , et al: Effect of femoral head size and surgical approach on risk of revision for dislocation after total hip arthroplasty: An analysis of 166,231 procedures in the Dutch Arthroplasty Register (LROI). Acta Orthop 2017;88(4):395–401. 28440704 10.1080/17453674.2017.1317515 PMC5499330

[9] De Martino I Triantafyllopoulos GK Sculco PK , et al: Dual mobility cups in total hip arthroplasty. World J Orthop 2014;5(3):180–187. 25035820 10.5312/wjo.v5.i3.180 PMC4095010

[10] Ezquerra L Quilez MP Perez MA , et al: Range of movement for impingement and dislocation avoidance in total hip replacement predicted by finite element model. J Med Biol Eng 2017;37(1):26–34. 28286463 10.1007/s40846-016-0210-4 PMC5325855

[11] Kostensalo I Junnila M Virolainen P , et al: Effect of femoral head size on risk of revision for dislocation after total hip arthroplasty: A population-based analysis of 42,379 primary procedures from the Finnish Arthroplasty Register. Acta Orthop 2013;84(4):342–347. 23799348 10.3109/17453674.2013.810518 PMC3768031

[12] Turppo V Sund R Sirola J , et al: Cross-validation of arthroplasty records between arthroplasty and hospital discharge registers, self-reports, and medical records among a cohort of 14,220 women. J Arthroplasty 2018;33(12):3649–3654. 30193880 10.1016/j.arth.2018.08.010

[13] Grambsch P Therneau T : Proportional hazards tests and diagnostics based on weighted residuals. Biometrika 1994;81:515–526.

[14] Therneau T : A package for survival analysis in S. R package version 2.38, 2015. Available at: http://cran.r-project.org/package=survival

[15] AOANJRR: Annual report 2016. Available at: https://aoanjrr.sahmri.com/fi/annual-reports-2016 (2016, accessed 20 September 2018).

[16] NJR: 14th annual report. Available at: http://www.njrcentre.org.uk/njrcentre/ReportsPublicationsandMinutes/tabid/85/Default.aspx (2017, accessed 20 September 2018).

[17] Higgins BT Barlow DR Heagerty NE , et al: Anterior vs. posterior approach for total hip arthroplasty, a systematic review and meta-analysis. J Arthroplasty 2015;30(3):419–434. 25453632 10.1016/j.arth.2014.10.020

[18] Mjaaland KE Svenningsen S Fenstad AM , et al: Implant survival after minimally invasive anterior or anterolateral vs. conventional posterior or direct lateral approach. J Bone Joint Surg Am 2017;99:840–847. 28509824 10.2106/JBJS.16.00494

[19] Peters RM van Beers LWAH van Steenbergen LN , et al: Similar superior patient-reported outcome measures for anterior and posterolateral approaches after total hip arthroplasty: Postoperative patient-reported outcome measure improvement after 3 months in 12,774 primary total hip arthroplasties using the anterior, anterolateral, straight lateral, or posterolateral approach. J Arthroplasty 2018;33(6):1786–1793. 29502965 10.1016/j.arth.2018.01.055

[20] Cebatorius A Robertsson O Stucinskas J , et al: Choice of approach, but not femoral head size, affects revision rate due to dislocations in THA after femoral neck fracture: Results from the Lithuanian Arthroplasty Register. Int Orthop 2015;39(6):1073–1076. 25512138 10.1007/s00264-014-2618-1

[bibr21-1457496920930617] Sköldenberg O Ekman A Salemyr M , et al: Reduced dislocation rate after hip arthroplasty for femoral neck fractures when changing from posterolateral to anterolateral approach. Acta Orthop 2010;81(5):583–587. 20860452 10.3109/17453674.2010.519170 PMC3214747

[22] Enocson A Hedbeck CJ Tidermark J , et al: Dislocation of total hip replacement in patients with fractures of the femoral neck: A prospective cohort study of 713 consecutive hips. Acta Orthop 2009;80(2):184–189. 19404800 10.3109/17453670902930024 PMC2823165

[23] Conroy JL Whitehouse SL Graves SE , et al: Risk factors for revision for early dislocation in total hip arthroplasty. J Arthroplasty 2008;23(6):867–872. 18534522 10.1016/j.arth.2007.07.009

[24] Tsikandylakis G Kärrholm J Hailer NP , et al: No increase in survival for 36-mm versus 32-mm femoral heads in metal-on-polyethylene THA. Clin Orthop Relat Res 2018;476(12):2367–2378. Available at: http://www.ncbi.nlm.nih.gov/pubmed/30260863 (accessed 17 October 2018). 30260863 10.1097/CORR.0000000000000508 PMC6259897

[25] Shah SM Walter WL Tai SM , et al: Late dislocations after total hip arthroplasty: Is the bearing a factor? J Arthroplasty 2017;32(9):2852–2856. 28529109 10.1016/j.arth.2017.04.037

[26] Hooper GJ Rothwell AG Stringer M , et al: Revision following cemented and uncemented primary total hip replacement. J Bone Joint Surg Br 2009;91(4):451–458. 19336803 10.1302/0301-620X.91B4.21363

[27] Jørgensen CC Kjaersgaard-Andersen P Solgaard S , et al: Hip dislocations after 2,734 elective unilateral fast-track total hip arthroplasties: Incidence, circumstances and predisposing factors. Arch Orthop Trauma Surg 2014;134(11):1615–1622. 25118616 10.1007/s00402-014-2051-3

