# Supplementary material for: Posterior approach, fracture diagnosis, and american society of anesthesiology class iii–iv are associated with increased risk of revision for dislocation after total hip arthroplasty: An analysis of 33,337 operations from the finnish arthroplasty register
Source: Scand J Surg. 2020 Jun 5;110(3):351–8. doi: 10.1177/1457496920930617 (PMC8551428; doi:10.1177/1457496920930617)
Supplement: sj-pdf-1-sjs-10.1177_1457496920930617 – Supplemental material for Posterior approach, fracture diagnosis, and american society of anesthesiology class iii–iv are associated with increased risk of revision for dislocation after total hip arthroplasty: An analysis of 33,337 operations from the finnish [file sj-pdf-1-sjs-10.1177_1457496920930617.pdf]

Appendices

Appendix 1. Kaplan–Meier plot of ASA class I vs. ASA class II in the multivariable model after stratification.

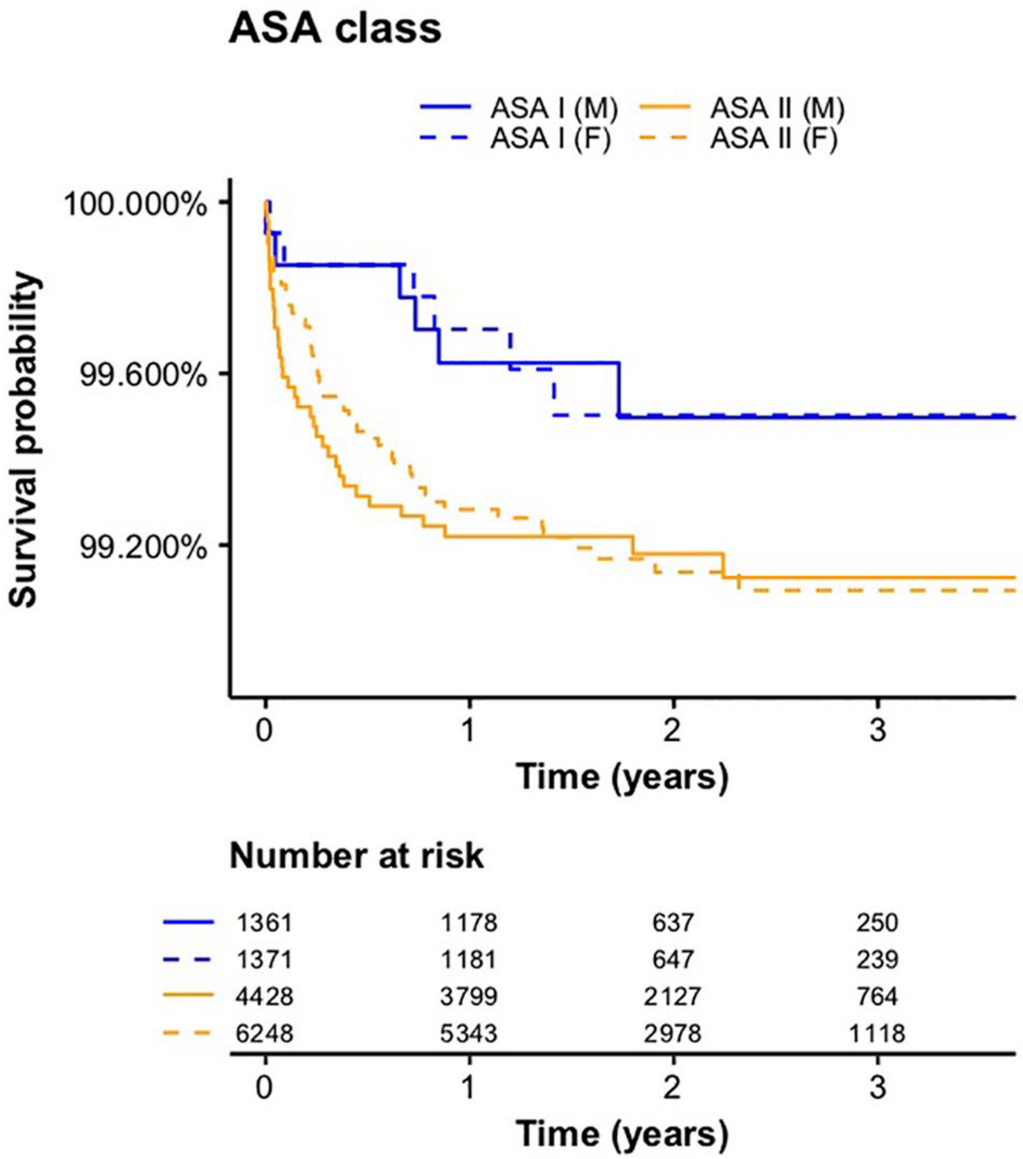

Appendix 2. Patient and surgical characteristics at the time of primary operation.

Values are mean S.D. for continuous variables and total number (%) for categorical variables.

Characteristic	Total			Dislocation			No dislocation		
	N available	N / mean	% / S.D.	N available	N / mean	% / S.D.	N available	N / mean	% / S.D.
Number of hips	33337			264			33073		
Age (years)	33330			264			33066		

≤ 55		4507	(13.5)		29	(11.0)		4478	(13.6)
56–65		8333	(25.0)		55	(20.8)		8278	(25.0)
66–75		12399	(37.2)		99	(37.5)		12300	(37.2)
≥ 76		8091	(24.3)		81	(30.7)		8010	(24.2)
Sex	33319			264			33055		
Male		14317	(43.0)		103	(39.0)		14214	(43.0)
Female		19002	(57.0)		161	(61.0)		18841	(57.0)
ASA physical status classification	32697			261			32436		
ASA I		4013	(12.3)		16	(6.1)		3997	(12.3)
ASA II		16117	(49.3)		112	(42.9)		16005	(49.4)
ASA III–IV		12567	(38.4)		133	(51.0)		12434	(38.3)
Body mass index (kg/m2)	30045			239			29806		
< 25		8345	(27.8)		68	(28.5)		8277	(27.8)
25–30		12309	(41.0)		100	(41.8)		12209	(40.9)
> 30		9391	(31.2)		71	(29.7)		9320	(31.3)
Preoperative diagnosis	32315			255			32060		
Primary osteoarthritis		27965	(86.6)		192	(75.3)		27773	(86.6)
Fracture		1366	(4.2)		33	(12.9)		1333	(4.2)
Other		2984	(9.2)		30	(11.8)		2954	(9.2)
Hospital volume	33333			264			33069		
Low		13042	(39.1)		86	(32.6)		12956	(39.2)
Medium		10279	(30.9)		87	(32.9)		10192	(30.8)
High		10012	(30.0)		91	(34.5)		9921	(30.0)
Level of education (surgeon)	29853			237			29616		
Orthopedic specialist		28438	(95.3)		223	(94.1)		28215	(95.3)
Resident		1415	(4.7)		14	(5.9)		1401	(4.7)
Level of education (assistant)	29003			232			28771		
Orthopedic specialist		2877	(9.9)		25	(10.8)		2852	(9.9)
Resident		8162	(28.2)		66	(28.4)		8096	(28.2)
No		1189	(4.1)		6	(2.6)		1183	(4.1)
Other		16775	(57.8)		135	(58.2)		16640	(57.8)
Surgical approach	32652			260			32392		

Anterolateral (modified Hardinge)		6151	(18.8)		22	(8.5)		6129	(18.9)
Posterior		26203	(80.3)		235	(90.4)		25968	(80.2)
Anterior (Smith–Peterson)		298	(0.9)		3	(1.1)		295	(0.9)
Intraoperative bleeding	31381			253			31128		
< 500 ml		21839	(69.6)		159	(62.8)		21680	(69.6)
≥ 500 ml		9542	(30.4)		94	(37.2)		9448	(30.4)
Duration (min)	27645	78	(28)	220	79	(30)	27425	78	(28)
Anesthesia (spinal)	32604			260			32344		
No		2485	(7.6)		23	(8.8)		2462	(7.6)
Yes		30119	(92.4)		237	(91.2)		29882	(92.4)
Anesthesia (epidural)	32604			260			32344		
No		31813	(97.6)		247	(95.0)		31566	(97.6)
Yes		791	(2.4)		13	(5.0)		778	(2.4)
Anesthesia (general)	32604			260			32344		
No		30072	(92.2)		239	(91.9)		29833	(92.2)
Yes		2532	(7.8)		21	(8.1)		2511	(7.8)
Anesthesia (nerve block)	32604			260			32344		
No		32598	(100.0)		260	(100.0)		32338	(100.0)
Yes		6	(0.0)		0	(0.0)		6	(0.0)
Anesthesia (LIA)	32604			260			32344		
No		26367	(80.9)		226	(86.9)		26141	(80.8)
Yes		6237	(19.1)		34	(13.1)		6203	(19.2)
Complications during surgery (fracture)	31395			249			31146		
No		30993	(98.7)		246	(98.8)		30747	(98.7)
Yes		402	(1.3)		3	(1.2)		399	(1.3)
Previous operation to the same joint	28071			220			27851		
No		27466	(97.8)		212	(96.4)		27254	(97.9)
Yes		605	(2.2)		8	(3.6)		597	(2.1)
Fixation	30150			247			29903		
Cementless		18655	(61.9)		133	(53.8)		18522	(61.9)

Cemented	3008 (10.0)	33 (13.4)	2975 (10.0)
Hybrid	6837 (22.7)	69 (27.9)	6768 (22.6)
Reverse hybrid	1650 (5.4)	12 (4.9)	1638 (5.5)
Bearing	25107	210	24897
Metal-on-UHXLPE	12652 (50.4)	132 (62.9)	12520 (50.3)
Ceramic-on-ceramic	2786 (11.1)	13 (6.2)	2773 (11.1)
Ceramic-on-UHXLPE	7063 (28.1)	51 (24.3)	7012 (28.2)
Ceramized metal-on-UHXLPE	1445 (5.8)	3 (1.4)	1442 (5.8)
Other	1161 (4.6)	11 (5.2)	1150 (4.6)
Oblique liner	30228	228	30000
No	23658 (78.3)	173 (75.9)	23485 (78.3)
Yes	6570 (21.7)	55 (24.1)	6515 (21.7)
Femoral head size (mm)	32452	252	32200
32	7836 (24.1)	87 (34.5)	7749 (24.1)
36	23958 (73.8)	158 (62.7)	23800 (73.9)
>36	311 (1.0)	4 (1.6)	307 (0.9)
28	347 (1.1)	3 (1.2)	344 (1.1)

Appendix 3. Univariate analysis of all predictors with incident of revision for dislocation.

Characteristic	Hazard ratio	95% CI	p-value
Age			0.05
≤ 55	Reference		
56–65	1.0	(0.7 – 1.6)	0.9
66–75	1.2	(0.8 – 1.9)	0.3
≥ 76	1.6	(1.0 – 2.4)	0.04
ASA physical status classification			<0.001
ASA I	Reference		
ASA II	1.8	(1.0 – 3.0)	0.03
ASA III – IV	2.7	(1.6 – 4.5)	<0.001
Body mass index (kg/m2)			0.9
< 25	Reference		
25 – 30	1.0	(0.7 – 1.3)	0.9
> 30	0.9	(0.7 – 1.3)	0.7
Preoperative diagnosis			<0.001
Primary osteoarthritis	Reference		
Fracture	3.6	(2.5 – 5.2)	<0.001
Other	1.5	(1.0 – 2.1)	0.05
Hospital volume			0.06
Low	Reference		

Medium	1.3 (1.0 – 1.8)	0.08
High	1.4 (1.0 – 1.9)	0.03
Level of education (surgeon)		
Orthopedic specialist	Reference	
Resident	1.2 (0.7 – 2.1)	0.4
Level of education (assistant)		0.6
Orthopedic specialist	Reference	
Resident	1.0 (0.6 – 1.5)	0.9
No	0.6 (0.2 – 1.5)	0.3
Other	1.0 (0.6 – 1.5)	0.9
Surgical approach		<0.001
Anterolateral (modified Hardinge)	Reference	
Posterior	2.6 (1.7 – 4.1)	<0.001
Anterior (Smith – Peterson)	2.9 (0.9 – 9.6)	0.09
Intraoperative bleeding		
< 500 ml	Reference	
≥ 500 ml	1.3 (1.0 – 1.7)	0.04
Duration (min)	1.0 (1.0 – 1.0)	1.0
Anesthesia (spinal)		
No	Reference	
Yes	0.8 (0.5 – 1.3)	0.3
Anesthesia (epidural)		
No	Reference	
Yes	2.0 (1.2 – 3.6)	0.01
Anesthesia (general)		
No	Reference	
Yes	1.1 (0.7 – 1.7)	0.7
Anesthesia (LIA)		
No	Reference	
Yes	0.6 (0.5 – 0.9)	0.02
Complications during surgery (fracture)		
No	Reference	
Yes	0.9 (0.3 – 2.9)	0.9
Previous operation to the same joint		
No	Reference	
Yes	1.7 (0.8 – 3.4)	0.1
Fixation		0.02
Cementless	Reference	
Cemented	1.6 (1.1 – 2.4)	0.01
Hybrid	1.4 (1.1 – 1.9)	0.01
Reverse hybrid	1.4 (0.8 – 2.5)	0.3
Bearing		<0.001
Metal-on-UHXLPE	Reference	
Ceramic-on-ceramic	0.4 (0.2 – 0.7)	0.003
Ceramic-on-UHXLPE	0.7 (0.5 – 1.0)	0.03

Ceramized metal-on-UHXLPE	0.2	(0.1 – 0.6)	0.006
Other	0.9	(0.5 – 1.6)	0.7
Oblique liner			
No	Reference		
Yes	1.3	(0.9 – 1.7)	0.1
Femoral head size (mm)			0.002
32	Reference		
36	0.6	(0.5 – 0.8)	<0.001
>36	1.1	(0.4 – 3.1)	0.8
28	0.8	(0.2 – 2.4)	0.7

Appendix 4. Multivariable analysis of all predictors with incident of revision for dislocation. Only patients without any missing data for variables of interest (N=21,706) were included in the final multivariable models.

Characteristic	Hazard ratio	95% CI	p-value
Age (years)			0.8
≤55	Reference		
56–65	1.2	(0.7 – 2.2)	0.5
66–75	1.3	(0.7 – 2.4)	0.3
≥76	1.3	(0.7 – 2.6)	0.4
ASA physical status classification			0.09
ASA I	Reference		
ASA II	1.7	(0.9 – 3.3)	0.09
ASA III – IV	2.0	(1.0 – 3.9)	0.04
Preoperative diagnosis			<0.001
Primary osteoarthritis	Reference		
Fracture	3.0	(1.9 – 4.7)	<0.001
Other	1.4	(0.9 – 2.2)	0.2
Surgical approach			<0.001
Anterolateral (modified Hardinge)	Reference		
Posterior	3.1	(1.7 – 5.5)	<0.001
Anterior (Smith – Peterson)	3.6	(1.0 – 13.1)	0.05
Intraoperative bleeding			
< 500 ml	Reference		
≥ 500 ml	1.3	(0.9 – 1.7)	0.1
Anesthesia (spinal)			
No	Reference		
Yes	0.6	(0.2 – 2.1)	0.4
Anesthesia (epidural)			
No	Reference		
Yes	1.4	(0.6 – 3.1)	0.5
Anesthesia (general)			

No	Reference		
Yes	0.9	(0.2 – 3.4)	0.9
Anesthesia (LIA)			
No	Reference		
Yes	0.8	(0.5 – 1.3)	0.4
Fixation			0.9
Cementless	Reference		
Cemented	1.2	(0.7 – 2.3)	0.5
Hybrid	1.0	(0.7 – 1.5)	1.0
Reverse hybrid	1.2	(0.5 – 3.0)	0.7
Bearing			0.1
Metal-on-UHXLPE	Reference		
Ceramic-on-ceramic	0.6	(0.3 – 1.3)	0.2
Ceramic-on-UHXLPE	0.9	(0.6 – 1.3)	0.5
Ceramized metal-on-UHXLPE	0.3	(0.1 – 1.0)	0.06
Other	0.6	(0.2 – 1.3)	0.2
Femoral head size (mm)			0.004
32	Reference		
36	0.5	(0.4 – 0.7)	<0.001
>36	0.4	(0.0 – 2.6)	0.3
28	0.5	(0.1 – 3.4)	0.4
Hospital volume			0.1
Low	Reference		
Medium	1.4	(1.0 – 2.0)	0.07
High	1.3	(0.9 – 2.0)	0.2
